# Co-Administration of Lipid Nanoparticles and Sub-Unit Vaccine Antigens Is Required for Increase in Antigen-Specific Immune Responses in Mice

**DOI:** 10.3390/vaccines4040047

**Published:** 2016-12-06

**Authors:** Elizabeth A. Thoryk, Gokul Swaminathan, Steven Meschino, Kara S. Cox, Marian Gindy, Danilo R. Casimiro, Andrew J. Bett

**Affiliations:** 1 Infectious Diseases and Vaccines, Merck Research Laboratories, Merck & Co., Inc., Kenilworth, NJ 07033, USA; elizabeth_thoryk@merck.com (E.A.T.); gokul.swaminathan@merck.com (G.S.); kara_cox@merck.com (K.S.C.); DCasimiro@aeras.org (D.R.C.); 2 Medical Affairs, Global Human Health, Merck & Co., Inc., Kenilworth, NJ 07033, USA; steven_meschino@merck.com; 3 Pharmaceutical Sciences, Merck Research Laboratories, Merck & Co., Inc., Kenilworth, NJ 07033, USA; marian_gindy@merck.com

**Keywords:** vaccines, lipid nanoparticles, formulation, alum, adjuvants

## Abstract

A vast body of evidence suggests that nanoparticles function as potent immune-modulatory agents. We have previously shown that Merck proprietary Lipid NanoParticles (LNPs) markedly boost B-cell and T-cell responses to sub-unit vaccine antigens in mice. To further evaluate the specifics of vaccine delivery and dosing regimens in vivo, we performed immunogenicity studies in BALB/c and C57BL/6 mice using two model antigens, Hepatitis B Surface Antigen (HBsAg) and Ovalbumin (OVA), respectively. To assess the requirement for co-administration of antigen and LNP for the elicitation of immune responses, we evaluated immune responses after administering antigen and LNP to separate limbs, or administering antigen and LNP to the same limb but separated by 24 h. We also evaluated formulations combining antigen, LNP, and aluminum-based adjuvant amorphous aluminum hydroxylphosphate sulfate (MAA) to look for synergistic adjuvant effects. Analyses of antigen-specific B-cell and T-cell responses from immunized mice revealed that the LNPs and antigens must be co-administered—both at the same time and in the same location—in order to boost antigen-specific immune responses. Mixing of antigen with MAA prior to formulation with LNP did not impact the generation of antigen-specific B-cell responses, but drastically reduced the ability of LNPs to boost antigen-specific T-cell responses. Overall, our data demonstrate that the administration of LNPs and vaccine antigen together enables their immune-stimulatory properties.

## 1. Introduction

Vaccine adjuvants are used to improve the potency of the immune response to co-administered antigens. The addition of an adjuvant is generally a requirement for recombinant subunit or peptide vaccines that—although considered to be safer than live attenuated vaccines—are poorly immunogenic [1,2] In addition to improving potency, adjuvants can enhance immunological memory and coverage, and allow for antigen sparing and fewer doses. A wide variety of natural and/or synthetic molecules and targeted delivery systems are being evaluated as adjuvants in preclinical and clinical studies [3,4]. These systems seem to have a unique ability to deliver antigen and adjuvant to the same cell, while targeting the immune system [5]. Understanding the mode of action of these novel adjuvants and delivery systems will aid in their development and regulatory approval [6]. Adjuvants have been found to potentiate immune responses by a number of different methods, including a depot effect to attract antigen presenting cells, activating pattern recognition receptors such as toll-like receptors (TLR) and nucleotide-binding oligomerization domain-like receptors (NOD), and inflammasome activation [7]. Currently, very few adjuvants are approved for use in licensed human vaccines. These include alum, MF59 (oil emulsion), AS03^®^ (squalene-based adjuvant system 03), and AS04^®^ (monophosphoryl lipid A, MPL + alum). However, these approved adjuvants do not always evoke the desired protective or durable immune responses against different target pathogens, highlighting the need to identify and develop new adjuvants. In particular, there is a critical need for the development of adjuvants that can induce strong CD8+ T cell responses. In addition, adjuvants that are able to induce strong immune responses in immunologically hypo-responsive populations such as the elderly, immunocompromised and pediatric populations, and chronically infected individuals, are badly needed [8].

Bioengineered nanoparticles—especially lipid nanoparticles (LNP)—have emerged as efficient bio-delivery vehicles that can be designed to improve the delivery and presentation of active agents to specific immune cells and/or intracellular targets [9,10]. Nanoparticles offer the ability to physically combine and co-deliver adjuvants and antigens to desired immune cell types or receptors of interest [11,12,13,14]. Moreover, nanoparticle delivery systems can limit untargeted exposure of biological agents in vivo and minimize systemic toxicities [15,16].

We recently reported that co-administration of specific ionizable lipid nanoparticles (LNPs) with Hepatitis B surface antigen (HBsAg), and Ovalbumin (OVA) significantly enhances antigen-specific B-cell, CD4+ T-cell, and CD8+ T-cell responses in BALB/c (HBsAg) and C57BL/6 mice (OVA) [17]. While some reports have characterized the advantage of using nanoparticles to encapsulate/deliver antigens and TLR agonists such as PolyIC to induce strong antigen-specific CD8+ T cell responses [18,19], our LNPs do not require the encapsulation of TLR agonists or antigen to stimulate strong antigen-specific immune responses [17]. However, the specific requirement for the co-administration of LNPs and antigen and the specific formulation characteristics that dictate the successful generation of strong antigen-specific immune responses by LNPs were not evaluated.

In this study, using our established mouse immunogenicity model, we explored the requirements of administrating vaccine formulations containing LNPs and the recombinant antigens HBsAg and OVA, and evaluating antigen-specific B-cell and T-cell responses. We provide novel evidence that co-administration of LNP with recombinant antigen (such as HBsAg and OVA) at the same injection site and at the same time is required for the LNP′s ability to boost antigen-specific B-cell and T-cell responses. Additionally, we demonstrate that co-formulating LNPs with an aluminum adjuvant can reduce their potential to elicit strong T-cell responses.

## 2. Materials and Methods

### 2.1. Animals

**Figure 1:**
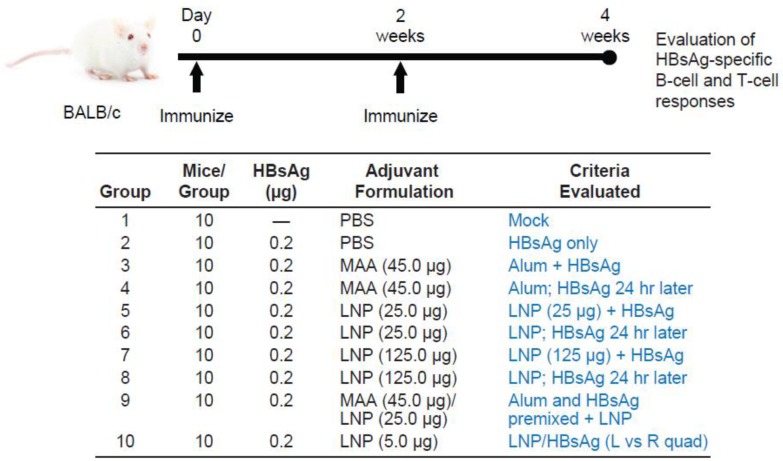
Immunization groups and vaccination regimes evaluated in BALB/c mice. The immunization regime and dosing schedule in BALB/c mice vaccinated with 0.2 μg of HBsAg formulated with LNPs and MAA is depicted. All vaccine formulations were administered a 100 μL dose (50 μL/quad) intramuscularly, except groups 4, 6, and 8, which received a 50 μL dose (25 μL/quad) at time 0 (adjuvant) and then again 24 h later (HBsAg). HBsAg: hepatitis B surface antigen; LNP: lipid nanoparticle; MAA: amorphous aluminum hydroxylphosphate sulfate; PBS: phosphate-buffered saline.

Female C57BL/6 and BALB/c mice—5–6 weeks old and pathogen free (Charles River Laboratories)—were maintained in accordance with Merck′s Institutional Animal Care and Use Committee (IACUC) protocols. For all experiments, intramuscular (IM) injections of 50 μL or 25 μL/dose per quadriceps were administered to either or both quadriceps muscles, with specified antigen and/or adjuvant. Detailed protocol outlines, including immunization dose regimes and timelines of sample collection are shown in Figure 1 and Figure 4.

### 2.2. Adjuvants and Vaccine Antigens

Lipid Nanoparticles were prepared at Merck (West Point, PA, USA) and characterized as previously described [17,20]. The LNPs are comprised of an ionizable amino lipid ((13Z,16Z)-*N*,*N*-dimethyl-3-nonyldocosa-13,16-dien-1-amine), distearoylphosphatidylcholine (DSPC), cholesterol, and poly(ethylene glycol)2000-dimyristoylglycerol (PEG2000-DMG), in a molar ratio of 58:30:10:2, respectively. Aluminum-based adjuvant AAHS (amorphous aluminum hydroxylphosphate sulfate, referred to as MAA), was manufactured at Merck & Co., Inc. (West Point, PA USA) as a proprietary formulation [21]. Recombinant Hepatitis B Surface Antigen (HBsAg) was manufactured at Merck & Co., Inc. and supplied at 40 μg/mL in saline. For all studies, HBsAg was dosed at 0.2 μg re-suspended in endotoxin-free phosphate buffered saline (PBS, with magnesium and calcium). Endotoxin-free Ovalbumin protein was purchased from Hyglos GmbH, supplied through Biovendor LLC; see figure legends for applicable doses of OVA diluted in PBS. Prior to vaccination, each antigen (at specified concentrations) was added to a vial containing the specific adjuvant (LNP or MAA), and mixed by inverting up and down five times. Then, using a sterile syringe, the specified volume of vaccination mixture was retrieved and administered to mice. Our group has recently published two papers where such detailed protocols are provided [17,22]. In addition, our group’s recent publication reported our characterization of antigen and adjuvant′s physio-chemical properties and stability [17].

### 2.3. Antigen-Specific Antibody Titers

Mice were injected twice with HBsAg or OVA +/− adjuvant at day 0 and day 14, and sera was collected 2 weeks post final immunization. HBsAg-specific antibodies were measured in duplicate by endpoint ELISA: MaxiSorp 96-well plates were coated with 0.75 μg/mL HBsAg in PBS. Goat anti-mouse immunoglobulin (Ig)G, anti-mouse IgG1, or anti-mouse IgG2a antibodies—all conjugated with horseradish peroxidase (HRP) (Southern Biotechnology 1010-05, 1070-05, 1080-05, Birmingham, AL, USA)—were used at 1:6000 dilution. Plates were developed using 3,3′,5,5′-Tetramethylbenzidine (TMB) (Virolabs, Sterling, VA, USA) and stopped with 0.2 N sulfuric acid, and absorbance was measured at 450 nm on a PerkinElmer Envision plate reader. All OVA assays were performed in triplicate: anti-OVA IgG and IgG2a antibody assay kits measured endpoint ELISA titers for OVA specific total IgG and IgG2a (Chondrex Inc., Redmond, WA, USA); mouse anti-OVA IgG1 EIA kit (Cayman chemicals, Ann Arbor, MI, USA) measured OVA specific IgG1 responses.

### 2.4. ELISpot Assays

Two weeks post final immunization, splenocytes were isolated from the spleens of vaccinated animals. Gamma interferon (IFN-γ)-secreting cells were determined using the enzyme-linked immunospot (ELISpot) assay, as previously described [23]. Briefly, multiscreen opaque plates (Millipore) coated with mouse anti-IFN-γ (clone AN18, MABTECH) were stimulated with 4 × 10^5^ cells/well and 2 μg/mL of HBsAg-specific peptide pools (15mer overlapping by 11, custom order JPT peptide solutions, Germany) or equal quantity of DMSO. Following incubation, plates were washed and probed with 100 μL per well of 1.25 μg/mL biotin-conjugated rat anti-mouse IFN-γ MAb (clone R4-6A2 MABTECH). The plates were developed using established procedures, and spots were enumerated with an imager (AID, Strassberg, Germany). The data was normalized to an input of 10^6^ cells.

### 2.5. T-Cell Intracellular Staining (ICS)

Spleens used in step 2.5 were used in ICS assays: one million splenocytes/well were incubated with 2 μg/mL of HBsAg-specific peptide pools (15mer overlapping by 11, custom order JPT peptide solutions, Germany) or equal quantity of DMSO in the presence of brefeldin A and anti-mouse CD28 and CD48b antibodies (BD Biosciences) for 6 h at 37 °C. Splenocytes were surface stained for anti-mouse-CD3 (BD cat #557596), CD4 (BD cat #550954), CD8 (BD cat #563068), permeabilized and stained for anti-mouse IFNγ (BD cat #557596). OVA affected splenocytes were incubated with 1 µg/mL of OVA major histocompatibility complex (MHC) class-I epitope peptide 257-264 (SIINFEKL) or OVA MHC class-II epitope peptide 323-339 (ISQAVHAAHAEINEAGR) purchased from Invivogen, San Diego, CA for 5 h at 37 °C. Splenocytes were surface stained for anti-mouse-CD3(#557596), CD4(#550954), CD8(#563068), permeabilized, and stained for anti-mouse IFNγ (BD cat #557596). A FACS LSRII flow cytometer read fixed samples (BD Biosciences), and data was analyzed using FloJo software (Treestar Inc., Ashland, OR, USA).

### 2.6. Statistical Analysis

A one-way ANOVA test with Bonferroni′s multiple comparison post hoc test or an unpaired, two-tailed, Student′s *t*-test using GraphPad Prism Software (GraphPad, San Diego, CA, USA) was used to determine statistical significance. Data is expressed as mean ± standard error of the mean (SEM). Differences between treatment groups and controls were considered statistically significant at ** *p* < 0.001.

## 3. Results

### 3.1. Requirement for Coadministration of HBsAg with LNP for Antigen-Specific B-Cell Responses

We first utilized HBsAg as an example of a well-established, clinically relevant viral vaccine antigen to assess the formulation and dosing conditions required to induce robust immune responses. BALB/c mice (*n* = 10/group) were immunized intramuscularly with 0.2 μg of HBsAg formulated in PBS (group 2) or adjuvant, as outlined in Figure 1. A prime/boost vaccination regime was followed for all vaccine formulations evaluated, with each dose injected two weeks apart. LNP doses of 25 μg (group 5) and 125 μg (group 7) were evaluated in comparison with well-established vaccine adjuvant amorphous aluminum hydroxylphosphate sulfate (MAA) (45 μg; group 3), as well as a formulation containing both LNP and MAA together (group 9). These formulations were prepared following our traditional method of mixing HBsAg with LNP or MAA (pre-incubated 30 min) just prior to injection into each quadriceps muscle. To prepare the formulation containing HBsAg + MAA + LNP, HBsAg was first pre-incubated for 30 min with MAA and then mixed with 25 μg of LNP prior to administration (group 9).

To assess the effect of timing of administration of the various components, LNP or MAA was injected into the quadriceps first (day 0 or 14), and then HBsAg was injected at the same site in the quadriceps 24 h later (groups 4, 6, and 8). In addition, we evaluated the requirement to administer the adjuvant and antigen to the same location by injecting LNP into the left quadriceps and HBsAg into the right quadriceps at the same time, day 0 and day 14 (group 10). The vaccines were given at 50 μL/quad (when antigen and adjuvant were co-formulated, or when antigen and adjuvant were given in different quads on the same day) or 25 μL/quad (when adjuvant was given 24 h before antigen) so that the total volume administered to each quad was equivalent.

**Figure 2:**
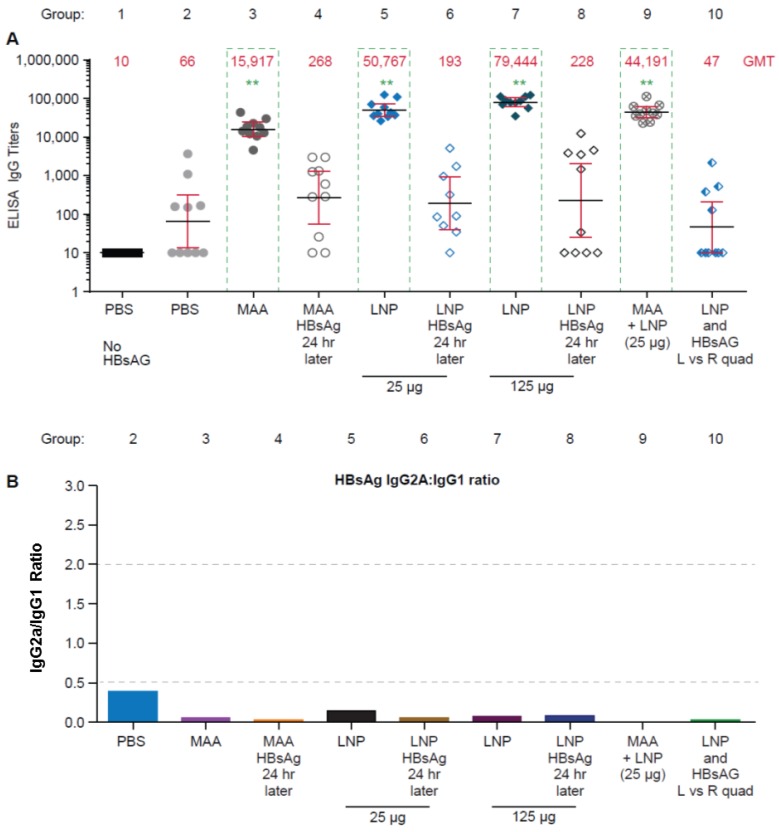
HBsAg-specific immunoglobulin (Ig)G responses in BALB/c mice. Lipid nanoparticles must be co-administered with HBsAg to enhance Th2 type specific B-cell responses in BALB/c mice. (**A**) Total IgG responses to HBsAg (individual animals); (**B**) IgG2a:IgG1 ratio to HBsAg determined by IgG2a and IgG1 ELISA (pooled serum). Mice were vaccinated with HBsAg in the following formulations: with LNP (groups 5 and 7), with MAA (group 3), with LNP after HBsAg binding with MAA (group 9), differentially administered LNP and HBsAg to left or right quadriceps muscle (group 10), or HBsAg administered 24 hours after LNP or MAA to the same site (group 6 and 8, or 4, respectively). ELISA assays were performed with sera collected at 2 weeks post-dose 2, in duplicate, from 10 mice/group. ** *p* < 0.001 denotes statistical significance compared to PBS + HBsAg group determined by one-way ANOVA.

Two weeks after the final dose, sera was collected and HBsAg-specific antibody responses were determined by an end-point dilution ELISA. Statistically significant increases in HBsAg-specific total IgG titers were observed in mice that received MAA + HBsAg (group 3), LNP + HBsAg (group 5 and 7), and MAA + LNP + HBsAg (group 9), as compared to the group that received PBS + HBsAg (group 2) (*p* < 0.001) (Figure 2A). Interestingly, responses were not observed when HBsAg was administered 24 h after the LNP (group 6 and 8) or MAA (group 4), or if the HBsAg and adjuvants were administered in different quadriceps muscles (group 10).

To further characterize our total IgG results, we measured IgG1 and IgG2a specific titers following previously described methods [12]. The ratio of the IgG2a to IgG1 titers has been used as a method of evaluating the quality of antigen-specific immune responses, such that a ratio of IgG2a:IgG1 of >2 is considered to be a Th1-type response, and a ratio of <0.5 is considered to be a Th2-type response. Consistent with previously reported data [12], in BALB/c mice, MAA + HBsAg, LNP + HBsAg, and MAA + LNP + HBsAg resulted in a Th2 response profile with IgG2a/IgG1 ratios for all groups of <0.5 (Figure 2B). Overall, this data demonstrates that LNP and MAA must be co-administered with HBsAg to enhance serum IgG antibody responses to HBsAg sub-unit antigen, and that the response is a Th2 type immune response.

### 3.2. Requirement for Co-Administration of HBsAg with LNP for Antigen-Specific T-Cell Responses

**Figure 3:**
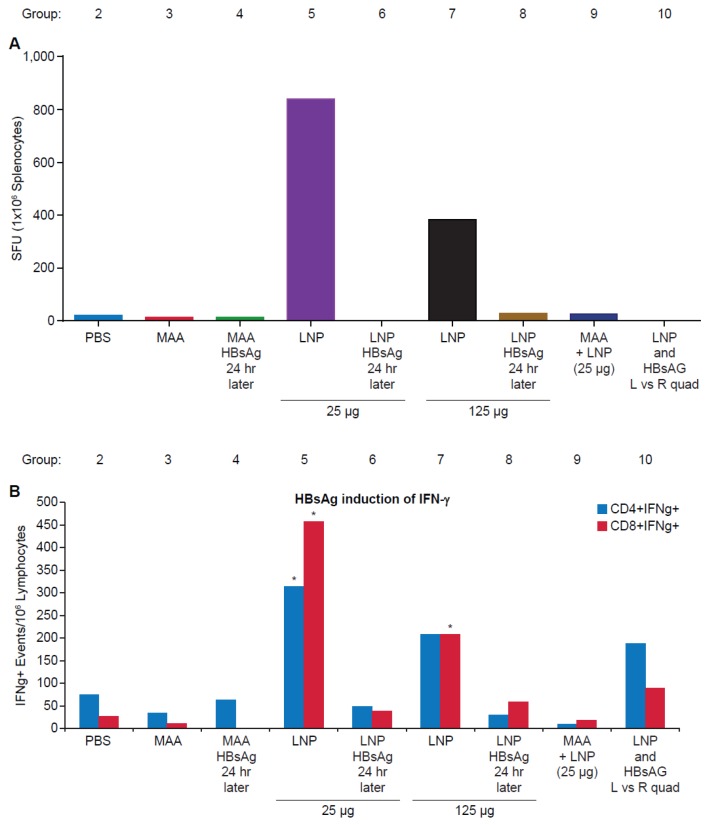
HBsAg-specific T-cell responses in BALB/c Mice. Spleens from five randomly selected mice out of ten immunized mice/group were collected, pooled, and processed two weeks after final vaccination. (**A**) The total HBsAg-specific T-cell response was evaluated using the IFN-γ ELISPOT assay to determine IFN-γ producing spots per million (SFU) splenocytes; or (**B**) HBsAg-specific CD4+ and CD8+ T cells that express IFN-γ were determined by Intracellular FACS assay (FACS ICS). ELISpot results are presented as the average value from pooled spleens, and the FACS ICS as T-cell events per million splenocytes.

Our previous studies have demonstrated that LNPs have the ability to enhance antigen-specific CD4+ and CD8+ T-cell responses. Having demonstrated that timing and location of administration of LNPs and HBsAg affects antigen-specific B-cell responses, we evaluated T-cell responses in the same vaccination groups. An ELISpot assay was utilized to test T-cell responses in mice two weeks after the final dose. Spleens were pooled from five randomly selected mice per group, processed, and stimulated with a pool of peptides covering the entire amino acid sequence of HBsAg. As shown in Figure 3A, significant antigen-specific IFNγ^+^ spot forming units (SFU) were observed only in the groups co-administered LNP and HBsAg, with the 25 μg LNP (group 5) generating a stronger response than 125 μg LNP (group 7). This data was further confirmed in an intracellular cytokine staining assay (ICS), which demonstrated statistically significant HBsAg-specific CD4 and CD8 T-cell responses in the group vaccinated with 25 μg LNP (group 5), and statistically significant CD8 T-cell responses in the group vaccinated with 125 μg LNP (group 7) (Figure 3B; ** *p* < 0.001). No elevation in T cell responses (ELISPOT or FACS ICS) were observed when the HBsAg and adjuvant were administered 24 h apart. When HBsAg and LNP were administered in the right and left quadriceps muscles, respectively, no responses were detectable by ELISPOT, and only a minimal T-cell response was detected by FACS ICS assay. Taken together, this data demonstrates a necessity to co-administer LNP and HBsAg to elicit robust cell-mediated immune responses.

### 3.3. Requirement for Co-Administration of OVA with LNP for Antigen-Specific B-Cell Responses

**Figure 4:**
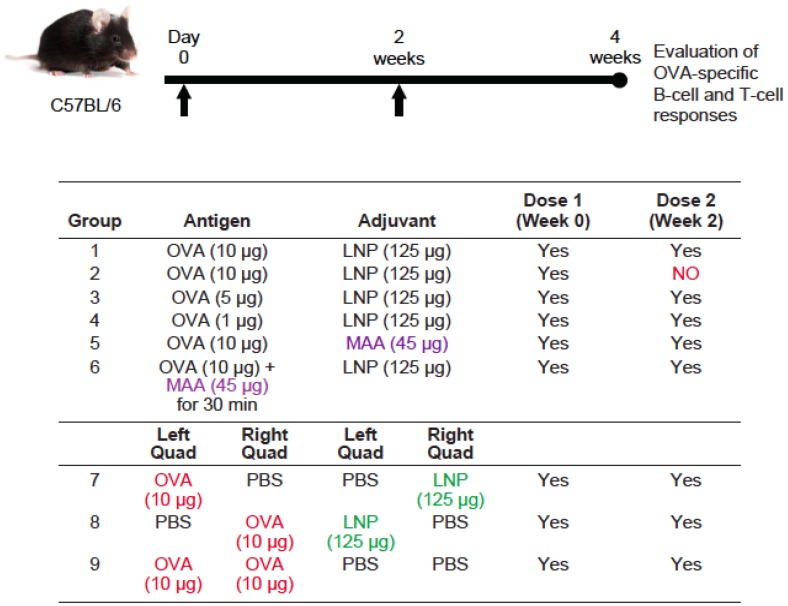
Immunization groups and vaccination regimes evaluated in C57BL/6 mice. OVA: ovalbumin. The immunization regime and dosing schedule for C57BL/6 mice vaccinated with 10 μg, 5 μg, or 1 μg of OVA antigen formulated with LNP and/or MAA are depicted. All immunizations were administered intramuscularly, 100 μL dose (50 μL/quad), except groups 7, 8, and 9, which received 50 μL dose (25 μL/quad).

To further validate the results achieved with HBsAg in BALB/c mice, we evaluated the administration requirements of LNPs and OVA antigen in C57BL/6 mice. C57BL/6 mice (*n* = 10/group) were immunized intramuscularly (50 μL/quad) with OVA, formulated in PBS (group 9) or adjuvant, as outlined in Figure 4. A prime/boost vaccination regime was followed for all vaccine formulations evaluated, except for group 2, which evaluated a single vaccine dose. We evaluated and compared the immune responses in animals that received a 10 μg dose of OVA formulated with LNP (125 μg) (group 1), MAA (45 μg; group 5), or with both MAA and LNP (group 6). We also evaluated 5 μg (group 3) and 1 μg (group 4) doses of OVA formulated with LNP (125 μg). These formulations were prepared following our traditional method of mixing OVA with LNP or MAA (pre-incubated 30 minutes) just prior to injection into each quadriceps muscle. To prepare the formulation containing OVA + MAA + LNP, OVA was first pre-incubated for 30 min with MAA, and then mixed with LNP prior to administration.

In addition, we evaluated the requirement to administer the adjuvant and antigen in the same location by injecting LNP into the left or right quadriceps and OVA into the opposite quadriceps, respectively (group 7 and 8). Two weeks after the final dose, sera was collected, and OVA-specific antibody responses were determined by end-point dilution ELISA.

**Figure 5:**
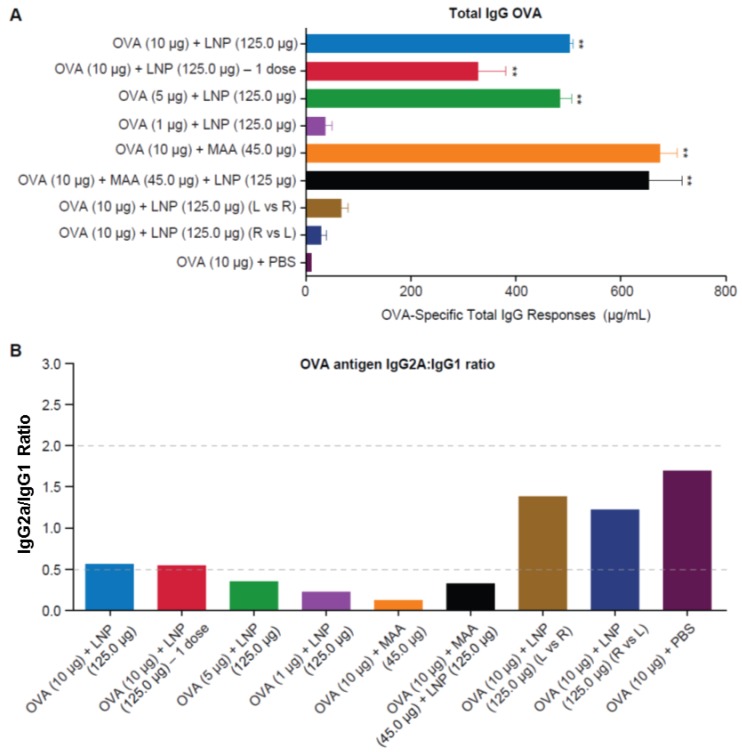
OVA specific total IgG responses in C57BL/6 mice. Lipid Nanoparticles must be co-administered with OVA antigen to enhance Th2 type specific B-cell responses in mice. (**A**) Total IgG responses to OVA antigen determined by ELISA (individual animals); (**B**) The IgG2a:IgG1 ratio to OVA determined by IgG2a and IgG1 ELISA (pooled serum). ELISA assays were performed with sera collected at 2 weeks post-dose 2, in duplicate, from 10 mice/group. Mice were vaccinated with OVA antigen alone (group 9) or formulated with LNP (groups 1–4), MAA (group 5), or LNP and MAA (group 6). Groups were also included the received HBsAg and LNP in opposite quadriceps muscles (groups 7 and 8). ** *p* < 0.001 denotes statistical significance compared to PBS group using one-way ANOVA or Student’s *t*-test.

As shown in Figure 5A, statistically significant increases in OVA-specific total IgG titers were observed in mice that received OVA + MAA, OVA + LNP, and OVA + MAA + LNP, as compared to the unadjuvanted PBS + OVA group (*p* < 0.001). Decreasing the dose of OVA from 10 μg (group 1) to 5 μg (group 3) did not impact the total IgG responses; however, decreasing the dose of OVA to 1 μg (group 4) reduced the antibody responses dramatically, such that they were similar to the HBsAg + PBS control group. Interestingly, administering one dose of 10 μg of OVA with LNP (group 2) was sufficient to generate a statistically significant OVA specific IgG response. As observed with HBsAg, when OVA and LNP were administered in opposite quadriceps muscles, there was a dramatic drop in OVA specific IgG titers. As was done for HBsAg, we measured IgG1 and IgG2a specific titers to assess the quality of the immune response elicited in the various vaccine groups. Consistent with the HBsAg experiments, vaccination of mice with OVA + LNP, OVA + MAA, or OVA + MAA + LNP resulted in a Th2-type response profile, with IgG2a/IgG1 ratios around 0.5 or less. In groups that received OVA formulated in PBS or those that received OVA and LNP in opposite quadriceps muscles, the ratios indicated a more balanced Th0-type response (IgG2a:IgG1 ratio between 0.5 and 2) (Figure 5B). A dose response was clear in the IgG2a/IgG1 ratio for the 10 μg, 5 μg, and 1 μg OVA and LNP groups (groups 1, 3, and 4). These data are in agreement with the data generated with HBsAg, and support the observation that LNP and MAA must be co-administered with antigen in the same location to elicit robust humoral immune responses.

### 3.4. Requirement for Co-Administration of OVA with LNP for Antigen-Specific T-Cell Responses

As evaluated with HBsAg, we investigated the requirement for coadministration of LNPs and OVA to the same injection site for elicitation of antigen-specific T-cell responses in C57BL/6 mice. Using the same groups of mice described above (Figure 4), intracellular expression of IFN-γ was assessed by flow cytometry. Two weeks after the final dose of the various vaccine formulations, spleens were pooled from five randomly selected mice per group. The spleens were processed and stimulated with known immunodominant OVA-specific CD4+ peptide (257–264) and CD8+ peptide (323–339) and stained for intracellular expression of IFN-γ. Please confirm the superscript.

**Figure A1:**
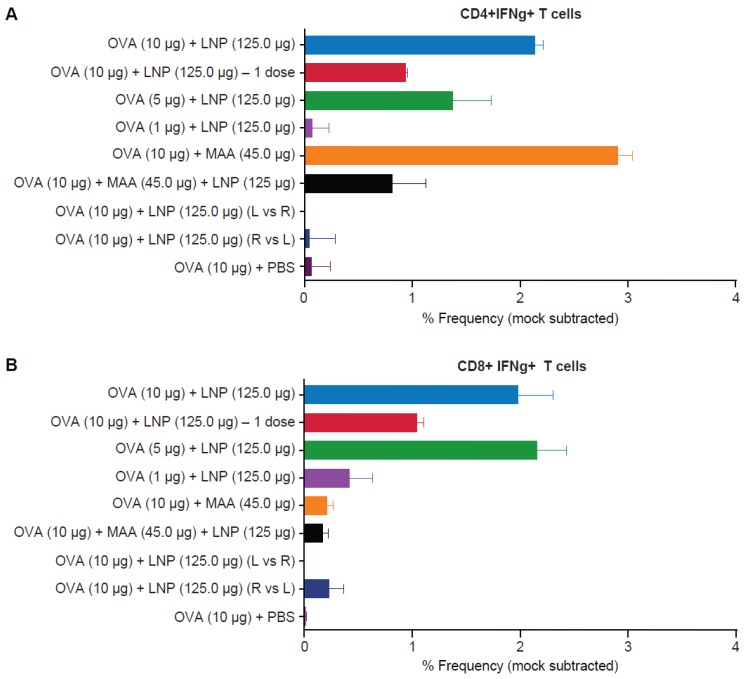
CD4 and CD8 specific T-cell responses to OVA in C57BL/6 mice. OVA antigen-specific CD4+ (**A**) and CD8+ (**B**) T cells that express gamma interferon (IFN-γ) were evaluated by intracellular FACS assay (FACS ICS). Bars represent T-cell events per million splenocytes from five randomly selected mice out of ten immunized mice/group.

Appendix Figure A1 depicts the percentage frequencies of OVA-specific CD4+ and CD8+ T cells expressing IFN-γ. Co-administration of OVA and LNP yielded strong OVA-specific CD4+ and CD8+ T-cell responses. Similar to what was observed for HBsAg, administration of antigen and adjuvant in opposite quadriceps muscles diminished the frequency of antigen-specific CD4+ and CD8+ T-cell responses. A titration of the CD4+ and CD8+ T-cell responses was seen moving from 10 μg to 1 μg. In addition, 1-dose of 10 μg OVA with LNP elicited CD4+ T-cell and CD8+ T-cell responses approaching half that of the 2-dose regimen. Co-administration of MAA with OVA generated a robust antigen-specific CD4+ T-cell responses, but no CD8+ T-cell responses. Co-formulating OVA with MAA and LNP resulted in CD4+ T-cell responses that were weaker then when OVA was formulated with each adjuvant separately. The CD8+ T-cell responses were also very weak in the MAA+LNP group, similar to what was seen with MAA alone.

## 4. Discussion

The field of nanoparticle systems as vaccine delivery platforms and adjuvants has expanded recently due to the abundance of research highlighting the effectiveness of such delivery systems—particularly for the development of sub-unit vaccines. Emerging evidence suggests that nanoparticle-based immune-modulators have promising potential applications in the development of novel cancer vaccines and cancer directed immune-therapies [24]. We recently reported novel ionizable lipid nanoparticle formulations that—when combined with sub-unit vaccine antigens—did not require co-administration of immune agonists to generate strong B-cell and T-cell responses, including CD8+ T-cell responses [17]. However, the specific vaccination regimes required for the LNPs to boost antigen-specific responses were not thoroughly evaluated. Some of the questions addressed in the studies reported here are: (i) whether it is necessary to administer the components of the vaccine at the same site and at the same time, (ii) if the components can be administered to the same site but at a different time interval, and (iii) the effect of combining LNPs and MAA in a co-administrated formulation.

Aluminum adjuvants are one of the best-characterized adjuvants; they have been shown to work through multiple mechanisms of action [25,26], including (i) inducing local inflammation (inflammasome activation), (ii) interaction with co-formulated recombinant proteins/antigens [27], (iii) enhancement of antigen retention at the site of injection (depot effect), and (iv) promotion of Th2 antibody responses. Aluminum has been demonstrated to have a synergistic effect when combined with MPL (TLR4 agonist) in the AS04 adjuvant formulation [28]. Furthermore, Didierlaurent et al. showed that in order to produce the optimal immune response, it was necessary to co-administer aluminum and MPL with their vaccine antigen [28]. Our previous work demonstrated our Merck LNP′s ability to generate robust B-cell and T-cell responses when formulated to contain TLR-9 agonist, when co-mixed with TLR-9 agonist or when the LNPs were used alone to adjuvant subunit antigens. However, this research did not investigate whether the LNPs required co-administration with vaccine antigens to elicit these responses or what effect aluminum (MAA) might have on LNP formulations. Aluminum adjuvants are known to generate robust humoral immune responses with acceptable safety profiles, but they are poor stimulators of T-cell-mediated immune responses [29]. Research from Saade et al. showed the ability of Advax^TM^ adjuvant (when substituted for alum) to enhance HBsAg-specific antibody and T-cell responses [30]. Therefore, we hypothesized that B-cell stimulating MAA combined with CD4+ and CD8+ stimulating LNP might generate a vaccine formulation that stimulates both the humoral and the cell-mediated immune pathways. Historically, the majority of our vaccine investigations have utilized the intra-muscular route of administration, including marketed vaccines that contain aluminum adjuvants (MAA). Therefore, building on the wealth of data surrounding MAA-based vaccines, we chose to focus on investigating LNP and antigen formulation requirements using the intramuscular vaccination route of administration in well-established mice vaccination models using HBsAg and OVA as antigens.

The data presented in this study demonstrate that LNP and MAA—when individually formulated with antigen (HBsAg or OVA)—results in robust antigen-specific B-cell responses, as expected. Interestingly, we also found no statistically significant impact on antigen-specific B-cell responses when antigen was combined with both LNP and MAA. We also found that LNP and MAA largely results in a Th2-type immune response as measured by IgG1:IgG2a ratio, and the combination of these two adjuvants does not change the polarization pattern. It should be pointed out that when evaluating OVA-specific Th1/Th2 type B-cell responses, a IgG2a specific antibody was used. Unlike BALB/c mice, C57BL/6 mice express the Igh1-b allele that encodes for the IgG2c sub-class of antibody (and not IgG2a) and relies on the cross reactivity of the IgG2a-specific antibody for detection [31].

In contrast to the B-cell immune responses, the LNP-mediated boost in antigen-specific CD4+ and CD8+ T cell responses were dramatically reduced in the presence of MAA. This observation was true for both HBsAg and OVA antigen in BALB/c and C57BL/6 mice, respectively. We speculate that the reduction in the cell-mediated immune response is from aluminum binding too tightly to antigen in the mixture, such that the avidity of aluminum–antigen binding might enable the depot effect and the generation of antibodies, but interfere with antigen degradation and antigen presentation to T-cells. This is in agreement with several previous reports that have suggested that the strength of aluminum adjuvant′s binding to antigen dictates the generation of T-cell responses [5,32,33]. We propose that this antigen presentation step is crucial to LNP adjuvanting properties. Newer data suggest that antigen administered with aluminum adjuvant travels to the lymph node in a soluble form [34]; this might be sufficient for B-cell receptor recognition and the induction of B-cell responses, but not for the T-cell responses that are dependent on antigen processing and presentation. In line with this observation, we speculate that an antigen and MAA pre-incubation step of 30 min prior to mixing with LNP leads to an enhanced B-cell response, but a dampened T-cell response. As described, after 30 min of mixing MAA and antigen, LNPs were added prior to immunization. However, the physio-chemical mechanisms by which the LNPs directly interact with MAA or other aluminum adjuvants, and how this interaction might impact the observed antigen-specific responses will require future exploration.

The necessity of co-administration of the antigen and adjuvant was assessed by injecting antigen (HBsAg or OVA) and adjuvant (LNP or MAA) either co-mixed together or separately into opposite quadriceps muscles. In the case of HBsAg, the antigen was administered into the left quadriceps and LNP into the right quadriceps, while in the case of OVA, both quadriceps muscle combinations were assessed. Separating the injection site of the antigen and adjuvant completely negated the immune stimulatory properties of the LNPs for all the immune parameters assessed (B-cell, CD4+ T-cell, or CD8+ T-cell). The necessity of administering the antigen and adjuvant to the same location at the same time was assessed by injecting adjuvant first, followed 24 h later by antigen (HBsAg) in the same quadriceps. Saade et al. demonstrated that injection of aluminum adjuvant or Advax™ 24 h prior to or after HBsAg injection or in different limbs of mice resulted in significant modulation of HBsAg-specific immune responses [30]. In agreement with these findings, we found that location-differentiated administration, and administering the adjuvant 24 h prior to the antigen, completely negated the immune stimulatory properties of LNPs and MAA for all the immune parameters assessed (B-cell, CD4+ T-cell, or CD8+ T-cell).

It is yet to be determined if LNP vs. LNP and MAA (when combined with antigens) have differential outcomes in their ability to (i) impact myocytes upon intra-muscular injection, (ii) induce cytokines/chemokines, (iii) recruit specific innate immune cells, and (iv) modulate the trafficking of antigen-containing immune cells to the lymph node. It is possible that MAA enhances the LNP′s ability to activate B-cells or the recognition of antigen by the B-cells (or vice-versa). However, if LNP-mediated uptake of antigen by signature antigen presenting cells such as dendritic cells (DCs) is compromised in the presence of MAA (possibly due to increased neutrophil recruitment, etc.), or impacts antigen presentation, it might lead to diminished T-cell responses. Furthermore, specific innate immune pathways that may be activated by LNP, and how inclusion of MAA affects these pathways, are also unknown. Current and future studies aim at investigating all of the aforementioned hypotheses, and are an active area of interest to uncover LNPs’ mechanism of action.

Overall, we provide a comprehensive evaluation of LNP-containing vaccine formulations that highlights a critical need for careful evaluation of the vaccine regimen required to boost antigen-specific immune responses. We provide evidence that understanding the formulation requirement (i.e., co-mixing of antigens and the potential inclusion of additional adjuvants such as aluminum) may favor the generation of B-cell responses, but affect T-cell responses. Lastly, we provide evidence that clearly indicates the requirement for administration of sub-unit antigen with LNP at the same site, and at the same time. We believe that such detailed understanding of the formulations and vaccination factors that ultimately influence immune responses to LNP-based vaccines will guide further improvements to this vaccine model and support regulatory acceptance of LNP-containing vaccine formulations.

## 5. Conclusions

Bioengineered nanoparticles—especially lipid nanoparticles (LNP)—have emerged as efficient bio-delivery vehicles that can be designed to improve the delivery and presentation of active agents to specific immune cells and/or intracellular targets. We have demonstrated that co-administration of specific ionizable lipid nanoparticles (LNPs) with Hepatitis B surface antigen (HBsAg), and Ovalbumin (OVA) significantly enhances antigen-specific B-cell, CD4+ T-cell, and CD8+ T-cell responses in BALB/c (HBsAg) and C57BL/6 mice (OVA). The enhancement requires that the administration of sub-unit antigen with LNP occurs at the same site, and at the same time. In addition we found that the inclusion of additional adjuvants such as (aluminum) may favor the generation of B-cell responses, but negatively affect T-cell responses.
